# Terpenoids and their gene regulatory networks in *Opisthopappus taihangensis* ‘Taihang Mingzhu’ as detected by transcriptome and metabolome analyses

**DOI:** 10.3389/fpls.2022.1014114

**Published:** 2022-09-29

**Authors:** Hua Liu, Wendan Chen, Yuhong Chai, Wenchao Liu, Haixia Chen, Lei Sun, Xiaowei Tang, Chang Luo, Dongliang Chen, Xi Cheng, Fengjun Wang, Xiaohuan Yuan, Conglin Huang

**Affiliations:** ^1^ Beijing Academy of Agriculture and Forestry Sciences, Beijing Engineering Research Center of Functional Floriculture, Beijing, China; ^2^ Department of Food Science and Engineering, College of Biological Sciences and Biotechnology, Beijing Key Laboratory of Forest Food Processing and Safety, Beijing Forestry University, Beijing, China; ^3^ Beijing Liu Wenchao Institute of Summer Chrysanthemums Breeding Science and Technology, Beijing, China

**Keywords:** ‘Taihang Mingzhu’, transcriptome, RNA-Seq, metabolites, terpenoids

## Abstract

‘Taihang Mingzhu’ is the hybrid offspring of *Opisthopappus taihangensis*, and it has an excellent characteristic of whole-plant fragrance. At present, the genes and metabolites involved in the synthesis of its aromatic compounds are unknown because of the paucity of molecular biology studies on flowering in the *Opisthopappus* Shih genus. To elucidate the biosynthetic pathways of terpenoids, the main aromatic compounds in ‘Taihang Mingzhu’, we conducted transcriptome and metabolite analyses on its leaves and bud, inflorescences at the color-development, flowering, and full-bloom stages. A total of 82,685 unigenes were obtained, of which 43,901 were annotated on the basis of information at the protein databases Nr, SwissProt, KEGG, and COG/KOG (e-value<0.00001). Using gas headspace solid-phase microextraction chromatography – mass spectrometry (HS-SPME-GC/MS), 1350 metabolites were identified, the most abundant of which were terpenoids (302 metabolites). Analyses of the gene regulatory network of terpenoids in ‘Taihang Mingzhu’ identified 52 genes potentially involved in the regulation of terpenoid synthesis. The correlations between genes related to terpenoid metabolism/regulation and metabolite abundance were analyzed. We also extracted the essential oil from the leaves of ‘Taihang Mingzhu’ by hydrodistillation, and obtained 270 aromatic compounds. Again, the most abundant class was terpenoids. These results provide guidance for the extraction of essential oil from ‘Taihang Mingzhu’ leaves and flowers. In addition, our analyses provide valuable genetic resources to identify genetic targets to manipulate the aromatic profiles of this plant and other members the *Opisthopappus* Shih genus by molecular breeding.

## 1 Introduction

Members of the Asteraceae are among the most popular flower crops because of their rich color and unique fragrance (Sun et al., 2015). The genus *Opisthopappus* Shih is closely related to the genus *Chrysanthemum*. It has excellent characteristics such as tolerance to drought, shade, cold temperatures, and low-fertility soils, and is a wild germplasm resource unique to China (Chai et al., 2018). There are two species within the genus *Opisthopappus* Shih: *Opisthopappus taihangensis* and *Opisthopappus longilobus Shih*. These species grow on mountain slopes at an altitude of about 1,000 meters, mainly in the Taihang Mountains of Shanxi, Hebei, and Henan (Tang et al., 2012). Because of their unique habitat, low breeding rate, and few remnant populations, these species are listed as a Grade II endangered plants (He et al., 2012). To breed new superior species and produce new germplasm, hybrid breeding is one of the most widely used approaches. *O. taihangensis* plants are 10–15 cm tall, with a lavender or brown stem. The flowers are initially lavender, and then become white when they have fully bloomed. The whole plant including flowers and leaves produces a unique aroma. The flowering period of *O. taihangensis* is from June to September. *O. taihangensis* has a beautiful flower and aroma, but it is difficult to cultivate and propagate. *Chrysanthemum* is a closely related genus to *Opisthopappus* Shih, and *Chrysanthemum* plants are easy to propagate by cutting. Natural pollination has been used to generate hybrid progeny between *O. taihangensis* and *Chrysanthemum* with improved characteristics. *O. taihangensis* was used as the female parent and *Chrysanthemum* plants including *Chrysanthemum dichrum*, *Chrysanthemum lavandulifolium*, and some small *Chrysanthemum* species were used as the male parent for natural hybridization. These *Chrysanthemum* plants are 30–50 cm tall, with green stems and yellow flowers that are produced from July to September. One of these progenies is named ‘Taihang Mingzhu’. The whole plant of ‘Taihang Mingzhu’ is rich in essential oil, aromatic, 10–15 cm high, with green stems and pinnately divided leaves that are smooth and hairless on both sides. It has white flowers and blooms from June to September. The flower color, flowering period, and fragrance of ‘Taihang Mingzhu’ are similar to those of *O. taihangensis*, but its stem color is similar to that of *Chrysanthemum* plants. The leaf shape of ‘Taihang Mingzhu’ is between that of *Chrysanthemum* and *O. taihangensis*, and its leaf indentations are deeper than those of *Chrysanthemum* leaves, but shallower than those of *O. taihangensis* leaves (**Additional file 1**). When it rains in summer, the chrysanthemums growing on the plains usually die, while ‘Taihang Mingzhu’ cultivated under the same conditions continues to grow normally. In addition, *O. taihangensis* cuttings do not readily form roots, with a rooting rate of only 20%, while the rooting rate of ‘Taihang Mingzhu’ is as high as 92%. Thus, this plant has inherited many of the good characteristics of *O. taihangensis*, and it is easier to propagate and cultivate. Consequently, ‘Taihang Mingzhu’ is a very important germplasm resource in the genus *Opisthopappus* Shih and has broad prospects for development and utilization.

In recent years, research on *Opisthopappus* Shih has focused on its drought tolerance mechanism, genetic diversity, and population structure (Hong et al., 2006; Gu et al., 2019; Ye et al., 2021). High-throughput sequencing technology has been used to identify candidate genes in response to drought stress (Yang et al., 2020). The genetic diversity and population structure of the genus *Opisthopappus* Shih were analyzed using sequence-related amplified polymorphism molecular markers. The results showed that *O. taihangensis* and *O. longilobus* have high genetic diversity, and that the genetic diversity within populations varies between the two species. Genetic differentiation, gene flow, and geographic history may be influencing the genetic differentiation of the genus (Wang and Yan, 2013).

Aromatic substances are synthesized in all plant organs, including roots, stems, leaves, seeds, and fruit (Ramya et al., 2018). Aroma is one of the key traits of many flowering crops. Aroma compounds not only attract insects for pollination, but also act as antimicrobial agents against animal or insect species that can harm the plant (Giusto et al., 2010; Ramya et al., 2020). Quantitative aroma information is important to verify the authenticity of materials, as well as being an indicator of quality in production (Pineli et al., 2011). With the continuous development of techniques to extract and detect aromatic volatile compounds, more than 2000 volatile organic compounds have been identified from 90 plant families and 991 subspecies (Dunkel et al., 2009). Solid-phase microextraction (SPME) is a pretreatment technique for the extraction of volatile compounds. It allows for the rapid extraction of small amounts of volatiles and is suitable for floral fragrance analyses (Pawliszyn, 2001). According to their origin and biosynthetic pathways, aroma compounds can be classified into three main groups: terpenes, phenylpropanoids, and fatty acid derivatives (Knudsen et al., 1993).

The biosynthetic process of floral aroma compounds is complex and involves many enzymes in different tissues. Among the floral aroma compounds, terpenoids are the dominant group of volatile components, with a total of 556 compounds identified to date (Shalev et al., 2018; Huang et al., 2021). Terpenoids are biosynthesized by two different metabolic pathways: the mevalonate (MVA) pathway that mainly functions in the cytoplasm and endoplasmic reticulum, and generates secondary metabolites such as sterols, sesquiterpenes, and triterpenes; and the 2-C-methyl-D-erythritol-4-phosphate (MEP) pathway, which generates monoterpenes and diterpenes (Zhang et al., 2021). In the cytoplasm, the precursor substances for the synthesis of sesquiterpenes, triterpenes, and their derivatives, namely isopentenyl diphosphate (IPP), is formed by the mevalonate pathway (Heuston et al., 2012; Vranová et al., 2013). In the plastid, the precursors of monoterpenes, diterpenes, tetraterpenes, and their derivatives are formed by the MEP pathway. Those precursors, i.e., pyruvate and 3-phosphoglyceraldehyde, are intermediates of glycolysis or the C4 pathway, and undergo a series of reactions to form MEP, followed by dimethylpropyl diphosphate (DMAPP). The precursors are subsequently transformed into various terpenoids by terpene synthases (Ramya et al., 2019; Liao et al., 2016).

Biosynthetic pathways are a hot research topic in the area of floral aroma formation. Given the important value of aromatic compounds in flowers, understanding their biosynthesis at the enzymatic and genetic levels contributes to a better understanding of regulation of plant aroma formation (Chen et al., 2015). *Opisthopappus* Shih is a very important aromatic plant, but the composition of its aroma compounds and the regulatory mechanisms that control aroma formation remain unknown. The paucity of transcriptomic and metabolomic data for *Opisthopappus* Shih at different periods of inflorescence development has seriously hindered research on the molecular regulation of the accumulation of aromatic substances in this plant.

In this study, we analyzed the transcriptome and metabolome of ‘Taihang Mingzhu’ at different developmental stages. A combination of headspace solid-phase microextraction (HS-SPME) and gas chromatography-mass spectrometry (GC-MS) was used to systematically study the aroma composition of the flowers at each stage. These data, combined with the transcriptomic data, revealed details of gene regulatory network and candidate terpenoid biosynthetic genes. We detected strong correlations between certain genes and metabolites related to terpenoid biosynthesis. The results of this study shed light on the biological mechanism of the floral fragrance of ‘Taihang Mingzhu’. Our findings provide valuable information for further research on fragrance formation and its molecular regulation mechanism in *Opisthopappus* Shih, and identify candidate genes for genetic engineering to manipulate fragrance.

## 2 Results

### 2.1 Metabolomic analysis

**Figure 1:**
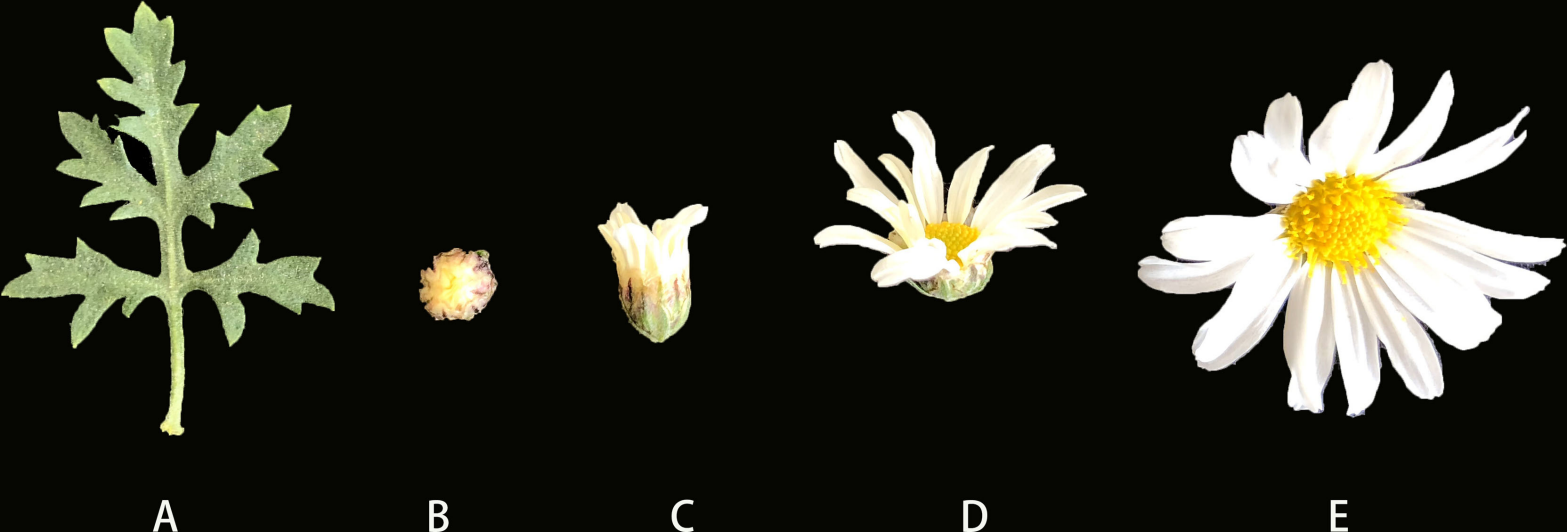
Plant material of ‘Taihang Mingzhu’ **(A)** Leaf. **(B)** Flower bud. **(C)** Inflorescence at color-development stage. **(D)** Inflorescence at flowering stage. **(E)** Inflorescence at full-bloom stage.

**Figure 2:**
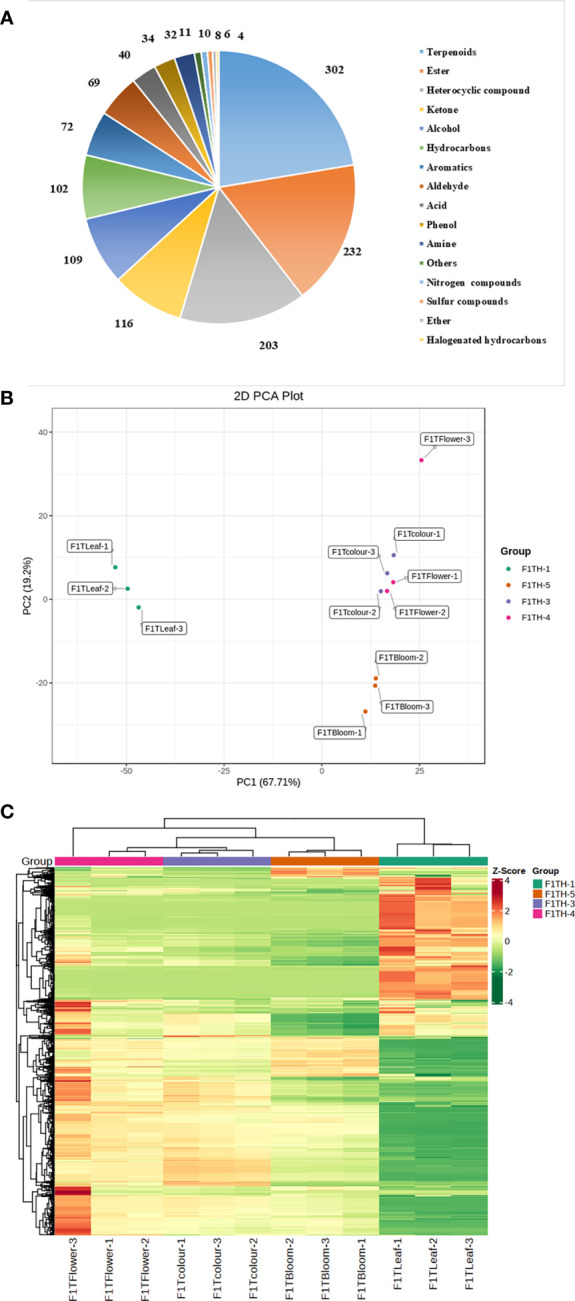
**(A)** Metabolite classification. **(B)** Principal component analysis plot of metabolomic samples. F1TH-1: leaf; F1TH-3, inflorescence at the color-development stage; F1TH-4, inflorescence at the flowering stage; F1TH-5, inflorescence at the full-bloom stage. **(C)** Cluster analysis of metabolomic samples.

To better understand the similarities and differences in metabolites in ‘Taihang Mingzhu’ flowers at different stages of development, we conducted HS-SPME-GC/MS analyses to identify and quantify the metabolites in leaves and the bud stage, inflorescences at color-development stage, flowering stage, and full-bloom stage (**Figure 1**). A total of 1350 metabolites were detected (**Figure 2A**, **Additional file 2**), comprising 302 terpenoids (22.37%), 232 esters (17.19%), 203 heterocyclic compounds (15.04%), 116 ketones (8.59%), 109 alcohols (8.07%), 102 hydrocarbons (7.56%), 72 aromatic hydrocarbons (5.33%), 69 aldehydes (5.11%), 40 acids (2.96%), 34 phenols (2.52%), 32 amines (2.37%), 11 others (0.81%), 10 nitrogenous compounds (0.74%), 8 sulfur compounds (0.59%), 6 ethers (0.44%), and 4 halogenated hydrocarbons (0.30%). Among them, terpenes were the main metabolites, both in terms of the number of different types and abundance. Terpenoids are the dominant class of secondary metabolites in members of the Asteraceae.

Principal component analysis (PCA) can reveal differences in overall metabolism among groups and the variation within groups. In the PCA plot (**Figure 2B**), the first principal component (PC1) explained 67.71% of the total variance and the second principal component (PC2) explained 19.2% of the total variance. The PCA analysis of the overall metabolites showed significant differences between the leaf and inflorescences of ‘Taihang Mingzhu’ at different developmental stages.

A cluster analysis (**Figure 2C**) revealed significant differences in volatile compounds in ‘Taihang Mingzhu’ inflorescences among all stages of development. In the inflorescences, the total metabolite content increased from the color-development stage to the flowering stage, and then decreased slightly from the flowering stage to the full-bloom stage. There were significant differences in the types of compounds between the inflorescences and the leaf. For example, the maximum number of terpenoids in the leaf was 292, but there were only four halogenated hydrocarbons. The metabolite with the highest relative content in the leaf was linalyl acetate, a terpenoid, while that in inflorescences at all periods was bicyclo[3.1.0]hexan-3-one, 4-methyl-1-(1-methylethyl)-, a different terpenoid. Information about these differences and variations in metabolites is important for elucidating the mechanisms of aroma formation during the development of ‘Taihang Mingzhu’.

### 2.2 Analysis of differentially accumulated metabolites

Orthogonal partial least squares discriminant analysis (OPLS-DA) was performed on all low molecular weight metabolites in the samples to screen for differential variables by removing uncorrelated differences. The values of R2X and R2Y were greater than 0.8 and that of Q2 was higher than 0.9 in all groups, indicating that the constructed model was valid and did not overfit.

Based on the OPLS-DA results, variable importance in projection (VIP) values combined with the difference multiplicity FC (fold-change) values were used to further screen for differentially accumulated metabolites. The criteria were VIP>1 and fold-change ≥ 2 or ≤ 0.5.

We detected differentially accumulated metabolites among different tissues of ‘Taihang Mingzhu’ (**Table 1**, **Additional file 3**). The comparison between the leaf (Leaf) and inflorescence at the color-development stage (Color) yielded the highest number of differentially accumulated metabolites (985), of which 660 were up-regulated and 325 were down-regulated in the inflorescence at the color-development stage compared with the leaf. The comparison between the inflorescence at the color-development stage (Color) and the inflorescence at the flowering stage (Flower) yielded the lowest number of differentially accumulated metabolites (87), of which 67 were up-regulated and 20 were down-regulated in inflorescence at the flowering stage compared with the color-development stage. The results of the pair-wise comparisons between other groups are shown in **Table 1**. These differentially accumulated metabolites may be important factors leading to the differences in aroma among ‘Taihang Mingzhu’ leaves and inflorescences at different developmental stages.

**Table 1:** Summary of the number of differentially accumulated metabolites among samples.

Group name	All sig diff	Down-regulated	Up-regulated
Leaf vs Color	985	325	660
Leaf vs Flower	910	276	634
Leaf vs Bloom	940	399	541
Color vs Flower	87	20	67
Color vs Bloom	218	189	29
Flower vs Bloom	169	155	14

The number of differentially accumulated metabolites is related to variations in the types and abundance of aromatic compounds such as terpenoids, esters, and alcohols, which ultimately form the aroma of ‘Taihang Mingzhu’. Terpenoids are synthesized by a series of enzymes through the MEP pathway or the MVA pathway.

We detected 1235, 1274, 1271, and 1274 aromatic compounds in the leaf and inflorescences of ‘Taihang Mingzhu’ at the color-development, flowering, and full-bloom stages, respectively. Among them, 292, 286, 286, and 286 were terpenes, respectively. Thus, there were more types of terpenoids in the leaf than in the inflorescences. The 16 leaf-specific terpenoids were 1,3,6-octatriene, 3,7-dimethyl-, (Z)-; beta-ocimene, 1,5-heptadien-4-ol, 3,3,6-trimethyl-; 2,4,6-octatriene, 2, 6-dimethyl-; 2-cyclohexen-1-one, 3-methyl-6-(1-methylethyl)-; (-)-carvone; (4R,4aS,6S)-4,4a-dimethyl-6-(prop-1-en-2-yl)-1,2,3,4,4a,5,6,7-octahydronaphthalene; 1,1,7,7a-tetramethyl-1a,2,6,7,7a,7b-hexahydro-1H-cyclopropa[a]naphthalene; naphthalene,1,2,4a,5,6,8a-hexahydro-4,7-dimethyl-1-(1-methylethyl)-, [1R-(1.alpha.,4a.alpha.,8a.alpha.)]-; spiro[4.5]dec-7-ene, 1,8-dimethyl-4-(1-methylethenyl)-, [1S-(1.alpha.,4.beta.,5.alpha.)]-; aromandendrene; carvenone; 2,6-octadienal, 3,7-dimethyl-, (E)-; cyclohexene, 3-(1,5-dimethyl-4-hexenyl)-6-methylene-, [S-(R*,S*)]-; isospathulenol; and hibaene. The 10 inflorescence-specific terpenoids were bicyclo[3.1.1]heptan-3-ol, 6,6-dimethyl-2-methylene-; cyclohexanol, 1-methyl-4-(1-methylethyl)-, cis-; 2H-pyran, 3,6-dihydro-4-methyl-2-(2-methyl-1-propenyl)-; p-mentha-1,5,8-triene; 2,6-dimethyl-1,3,5,7-octatetraene, E,E-; bicyclo[7.2.0]undec-4-ene, 4,11,11-trimethyl-8-methylene-,[1R-(1R*,4Z,9S*)]-; bornyl acetate; 2-cyclohexen-1-one, 2-hydroxy-3-methyl-6-(1-methylethyl)-; tricyclo[2.2.1.0(2,6)]heptane-3-methanol, 2,3-dimethyl-; and cyclopropanecarboxylic acid, 2,2-dimethyl-3-(2-methyl-1-propenyl)-, (1R-trans)-. Further analyses revealed that the types of terpenoids in the inflorescences were the same among different developmental stages, indicating that the synthesis of terpenoids in the inflorescences remained consistent from the color-development stage to the full-bloom stage.

Analyses of the relative contents of aromatic metabolites revealed that the leaf of ‘Taihang Mingzhu’ had the highest relative content of terpenoids (46.13%), followed by inflorescences at the flowering stage (38.78%), color-development stage (38.19%), and full-bloom stage (37.66%).

The substances with high relative contents in the leaf were linalyl acetate; epizonarene; bicyclo[2.2.1]heptane-2,5-dione, 1,7,7-trimethyl-; 7-oxabicyclo[4.1.0]heptane-2-one, 3-methyl- 6-(1-methylethyl)-; and 2,6-octadien-1-ol, 3,7-dimethyl-. The substances with relatively high contents in the inflorescences at the color-development stage were bicyclo[3.1.0]hexan-3-one, 4-methyl-1-(1-methylethyl)-; bicyclo[3.1.0]hexane, 4-methylene-1-(1-methylethyl)-; tricyclo[2.2.1.0(2,6)]heptane-3-methanol, 2,3-dimethyl-; benzene, 1-(1,5-dimethyl-4-hexenyl)-4-methyl-; and 2,6-dimethyl-1,3,5,7-octatetraene, E,E-. The substances with relatively high contents in inflorescences at the flowering stage were bicyclo[3.1.0]hexan-3-one, 4-methyl-1-(1-methylethyl)-; bicyclo[3.1.0]hexane, 4-methylene-1-(1-methylethyl)-; tricyclo[2.2.1.0(2,6)]heptane-3-methanol, 2,3-dimethyl-; benzene, 1-(1,5-dimethyl-4-hexenyl)-4-methyl-; and 2,6-dimethyl-1,3,5,7-octatetraene, E,E-. The substances with relatively high contents in the inflorescences at the full-bloom stage were bicyclo[3.1.0]hexan-3-one, 4-methyl-1-(1-methylethyl)-; bicyclo[3.1.0]hexane, 4-methylene-1-(1-methylethyl)-; tricyclo[2.2.1.0(2,6)]heptane-3-methanol, 2,3-dimethyl-; 2,6-dimethyl-1,3,5,7-octatetraene, E,E-; and thujone. The substances with relatively high contents in all inflorescences were bicyclo[3.1.0]hexane-3-one, 4 methyl-1-(1-methylethyl)-; thujone; and benzene, 1-(1,5-dimethyl-4-hexenyl)-4-methyl-; bicyclo[3.1.0]hexane, 4-methylene-1-(1-methylethyl)-.

### 2.3 Illumina sequencing and assembly

The transcriptomes of the leaf and inflorescences of ‘Taihang Mingzhu’ at the bud, color-formation, flowering, and blooming stages were sequenced using Illumina sequencing technology. The three leaf replicates yielded 31,551,295, 23,891,212, and 29,053,298 high-quality clean reads; those of buds yielded 24,002,761, 23,309,309, and 24,036,096 high-quality clean reads; those of inflorescences at the color-development stage yielded 25,860,836, 23,458,023, and 23,926,483 high-quality clean reads; those of inflorescences at the flowering stage yielded 26,747,007, 23,455,870, and 27,825,669 high-quality clean reads; and those of inflorescences at the blooming stage yielded 26,407,499, 24,961,746, and 24,734,570 high-quality clean reads.

After filtering the raw data to obtain clean data, a total of 82,685 unigene sequences were assembled using Trinity (Grabherr et al., 2011). The total size of the assembly was 67,629,859 bp, with a GC content of 39.2343% and an N50 of 1314 bp. The maximum read length was 13,054 bp, the minimum length was 201 bp, and the average length was 817 bp (**Table 2**). The unigene N50 length was greater than the average length, and the overall assembly quality was good.

**Table 2:** *De novo* assembly results.

Genes Num	GC %	N50 (bp)	MaxLength (bp)		MinLength (bp)	AverageLength (bp)	Totalassembled bases
82685	39.2343	1314	13054		201	817	67629859

### 2.4 Gene annotation and functional classification

**Figure 3:**
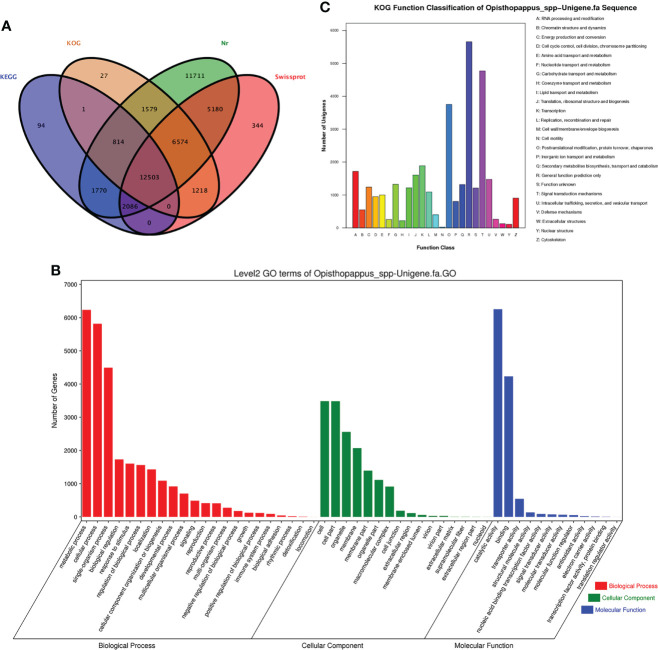
Gene annotation information. **(A)** Annotated Wayne diagram of four major databases of genes. **(B)** Histogram of gene ontology classifications. **(C)** Classification of eukaryotic homologous taxa (KOG) of *Opisthopappus*.

The 82,685 unigene sequences were searched against the protein databases Nr, SwissProt, KEGG, and COG/KOG (evalue<0.00001) by Blastx to obtain protein functional annotation information. Protein annotations were obtained for 43,901 unigenes. Among them, 42,217 unigenes were annotated in the Nr database, 27,905 unigenes were annotated in the Swissprot database, 22,716 unigenes were annotated in the KOG database, and 17,268 unigenes were annotated in the KEGG database, and 38,787 unigenes had no annotations (**Figure 3A**). The functional annotation of these genes laid the foundation for further analyses of terpenoid synthesis in ‘Taihang Mingzhu’.

GO annotations for unigenes were obtained using Blast2GO software (Conesa et al., 2005). After obtaining the GO annotation for each unigene, we used WEGO software to classify the GO functions of all unigenes (Ye et al., 2006) (**Figure 3B**). A total of 10,844 unigenes were annotated to 50 functional categories, including 22 subcategories in the biological process category, 12 subcategories in the molecular function category, and 16 subcategories in the cellular component category. In the biological process category, the subcategories with the most unigenes were “metabolic process” (GO:0008152, 6233 unigenes), “cellular process” (GO:0009987, 5816 unigenes), and “single-organism process” (GO:0044699, 4493unigenes). In the cellular component category, the subcategories with the most unigenes were “cell” (GO:0005623, 3486 unigenes), “cell par” (GO:0044464, 3484 unigenes), and “organelle” (GO:0043226, 2559 unigenes). In the molecular function category, the subcategories with the most unigenes were “catalytic activity” (GO:0003824, 6254 unigenes) and “binding” (GO: 0005488, 4242 unigenes). Among these three GO categories, the subcategories with the fewest unigenes were “locomotion” (GO:0040011, 1 unigene), “ translation regulator activity” (GO: 0045182, 1 unigene) and “nucleoid” (GO:0009295, 2 unigenes) (**Figure 2**).

The functions of the predicted proteins encoded by the genes were further analyzed on the basis of 25 functional categories in the KOG database. As shown in **Figure 3C**, the categories with the most unigenes were “General function prediction only” (5656 unigenes, 16.7%), “Signal transduction mechanisms” (4770 unigenes, 14.1%), and “Posttranslational modification, protein turnover, chaperones” (3751 unigenes, 11.1%). 11.1%). The categories with the fewest unigenes were “Extracellular structures” (125 unigenes, 0.4%), “Nuclear structure” (106 0.3%), and “Cell motility” (21 unigenes, 0.1%).

Next, KEGG annotation information was obtained to shed light on the biologically complex behavior of the genes in the transcriptome of ‘Taihang Mingzhu’ (Kanehisa, 2000). The unigenes were assigned to 134 pathways in these analyses (**Additional file 4**). The largest numbers of unigenes were assigned to “Metabolic pathways” (ko01100, 3441 unigenes) and “Biosynthesis of secondary metabolites” (ko01110, 1896 unigenes); and the smallest number of genes were assigned to “Glycosphingolipid biosynthesis – lacto and neolacto series” (ko00601, two unigenes), “Isoflavonoid biosynthesis” (ko00943, two unigenes), and “Anthocyanin biosynthesis” (ko00942, two unigenes) (**Table 3**).

**Table 3:** KEGG pathways enriched with ‘Taihang Mingzhu’ unigenes.

Pathway	Count	Ratio	Pathway ID
Metabolic pathways	3441	39.28%	ko01100
Biosynthesis of secondary metabolites	1896	21.64%	ko01110
Glycosphingolipid biosynthesis – lacto and neolacto series	2	0.02%	ko00601
Isoflavonoid biosynthesis	2	0.02%	ko00943
Anthocyanin biosynthesis	2	0.02%	ko00942

### 2.5 Differentially expressed genes among samples

In total, we detected 81,852 genes expressed in all samples of ‘Taihang Mingzhu’, accounting for 99.26% of the total number of genes. Pair-wise comparisons of different samples (leaf and inflorescences at four stages) were conducted to identify differentially expressed genes (DEGs). The criteria for DEGs were a false discovery rate (FDR) of <0.05 and log2 fold-change (|log2FC|) of >1.

A total of 16,503 DEGs were identified between the leaf and inflorescences at the bud stage, with 10,494 up-regulated and 6,009 down-regulated DEGs in the bud compared with the leaf. A total of 21,365 DEGs were identified between the leaf and inflorescences at the color-development stage, with 13,744 up-regulated and 7,621 down-regulated DEGs in the inflorescences compared with the leaf. A total of 20,371 DEGs were identified between the leaf and inflorescences at the flowering stage, with 7176 down-regulated DEGs in the inflorescences compared with the leaf. A total of 16,898 DEGs were identified between the leaf and the inflorescences at the full-bloom stage, with 10,674 up-regulated and 6,224 down-regulated DEGs in the inflorescences compared with the leaf.

**Figure 4:**
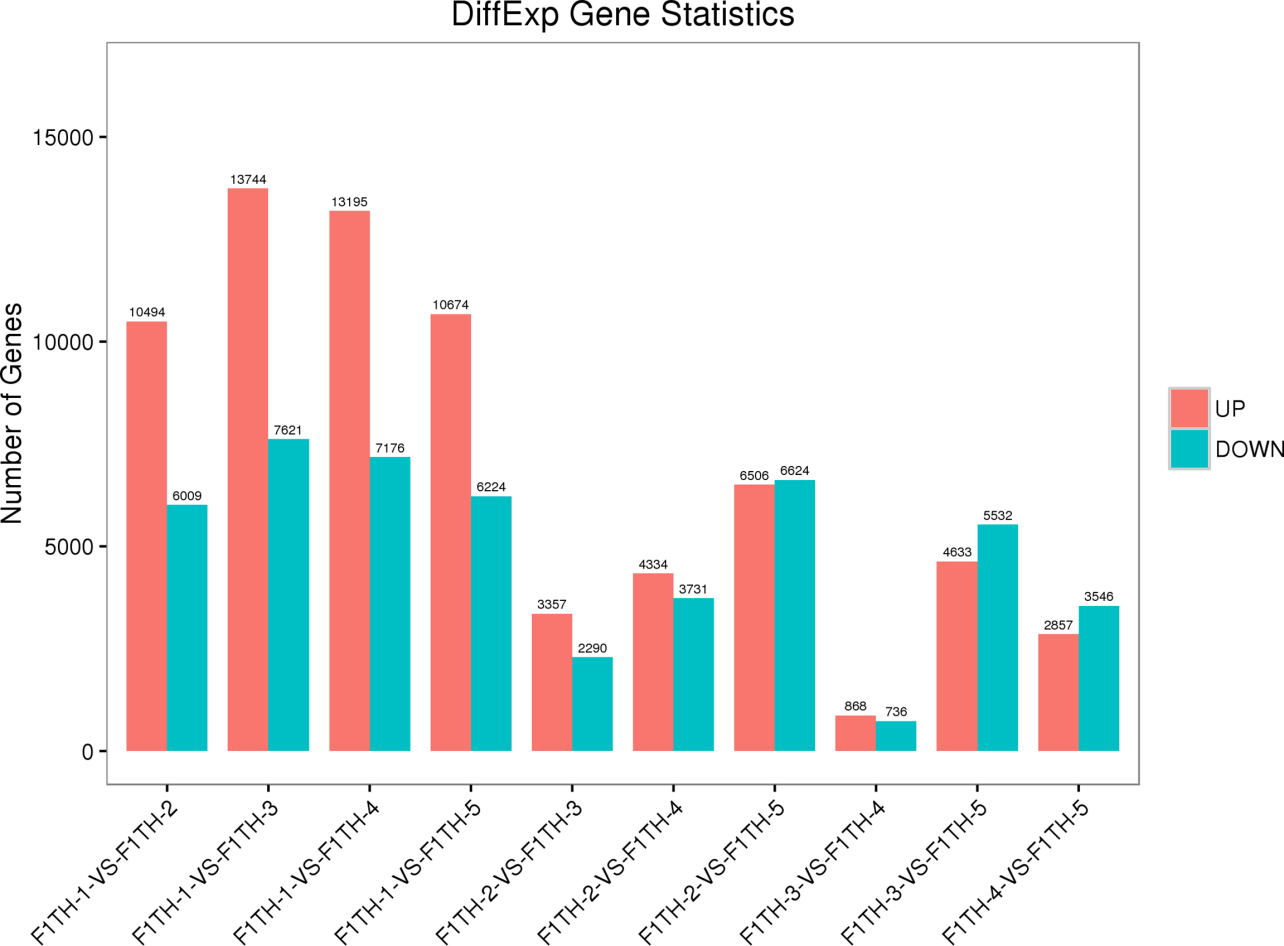
Numbers of differentially expressed genes among groups. F1TH-1, leaf; F1TH-2, bud stage; F1TH-3, inflorescence at the color-development stage; F1TH-4, inflorescence at the flowering stage; F1TH-5, inflorescence at the full-bloom stage.

A total of 5647 DEGs were identified between inflorescences at the bud stage and those at the color-development stage, with 3,357 up-regulated and 2,290 down-regulated DEGs in the inflorescences at the color-development stage compared with the buds. A total of 8065 DEGs were identified between inflorescences at the bud stage and those at the flowering stage, with 4334 up-regulated and 3,731 down-regulated DEGs in the inflorescences at the flowering stage compared with the buds. A total of 13,130 DEGs were identified between inflorescences at the bud stage and those at the full-bloom stage, with 6,506 up-regulated and 6,624 down-regulated DEGs in the full-boom flowers compared with the buds. A total of 1604 DEGs were identified between inflorescences at the color-development stage and those at the flowering stage, with 868 up-regulated and 736 down-regulated DEGs at the flowering stage compared with the color-development stage. A total of 10,165 DEGs were identified between inflorescences at the color-development stage and those at the full-bloom stage, with 4,633 up-regulated and 5,532 down-regulated DEGs at the full-bloom stage compared with the color-development stage. A total of 6403 DEGs were identified between inflorescences at the flowering stage and those at the full-bloom stage, with 2857 up-regulated and 3546 down-regulated at the flowering stage compared with the full-bloom stage (**Figure 4**).

These analyses showed that gene expression differed between the leaf and inflorescences. In ‘Taihang Mingzhu’, the highest number of DEGs was detected between the leaf and inflorescences at the color-development stage (13744 up-regulated, 7621 down-regulated), and the lowest number was detected between inflorescences at the color-development stage and those at the flowering stage (868 up-regulated, 736 down-regulated). The largest number of differentially accumulated metabolites was between the leaf and inflorescence at the color-development stage (985) and the smallest number was between the inflorescence at the color-development stage and the inflorescence at the flowering stage (87) (**Table 1**). These differences in gene expression may be related to differences in metabolite types and contents.

### 2.6 Differentially expressed genes related to terpenoid synthesis

**Figure 5:**
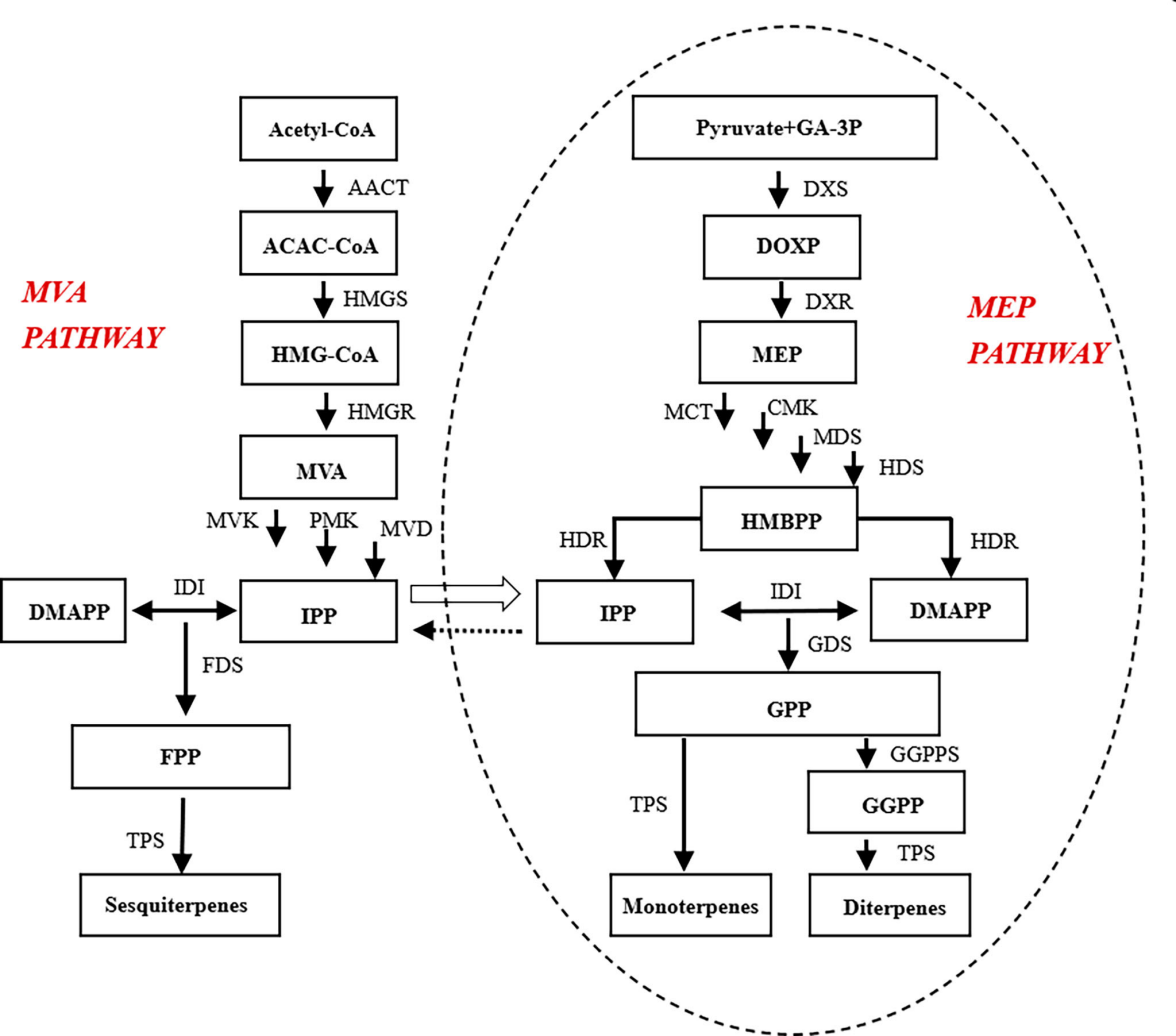
Important transcription factors in the terpenoid synthesis pathway of ‘Taihang Mingzhu’.

Plant floral aroma substances include terpenoids, phenyl/phenylpropane, and fatty acid derivatives (Mohd-Hairul et al., 2010). In ‘Taihang Mingzhu’, terpenoids are the main class of aromatic substances in terms of types and abundance, and are probably the main contributors to its floral fragrance. We analyzed the genes related to terpenoid synthesis and their regulatory networks (**Figure 5**), and determined the transcript levels of important regulatory and structural genes in terpenoid biosynthesis.

A total of 52 terpenoid synthesis-related genes were found in all samples (**Additional file 5**). The active expression of these genes regulated the synthesis of a rich variety of terpenoids that accumulated in the inflorescences of ‘Taihang Mingzhu’ at the color-development, flowering, and full-bloom stages, as well as in the leaf.

A pair-wise comparison between the leaf and inflorescence at the color-development stage revealed 19 differentially expressed terpenoid synthesis-related genes. Compared with the leaf, inflorescences at the color-development phase showed up-regulation of 16 genes: *OspAACT2, OspCMK2, OspDXS2, OspDXS3, OspFDS1, OspFDS2, OspGDS1, OspGDS3, OspHMGR1, OspHMGR2, OspHMGS2, OspMCT2, OspMVD1, OspMVK1, OspPMK1, OspTPS2. OspMVK1, OspPMK1*, and *OspTPS2*; and down-regulation of three genes: *OspHDS4, OspTPS1*, and *OspTPS7*.

In the comparison between the leaf and the bud stage, there were 18 differentially expressed terpenoid synthesis-related genes. Compared with the leaf, the bud showed up-regulation of 14 genes: *OspAACT2, OspCMK2, OspDXS2, OspDXS3, OspFDS1, OspGDS1, OspGDS3, OspHMGR1, OspHMGR2, OspHMGS2, OspMCT2, OspMVD1, OspMVK1*, and *OspTPS2*; and down-regulation of four genes: *OspHDS4, OspMDS1, OspTPS1*, and *OspTPS7*.

Comparing the leaf and inflorescences at the full-bloom stage, there were 19 differentially expressed terpenoid synthesis-related genes. Compared with leaf, the inflorescences at the full-bloom stage showed up-regulation of 15 genes: *OspAACT2, OspCMK2, OspDXR2, OspDXS2, OspDXS3, OspDXS4, OspGDS1, OspGDS3, OspGGDS1, OspGGDS2, OspHMGR1, OspHMGR2, OspHMGR2, OspMVK1. OspHMGS2, OspMVD1*, and *OspMVK1*; and down-regulation of four genes: *OspHDS4, OspHMGR3, OspTPS1*, and *OspTPS7*.

**Figure 6:**
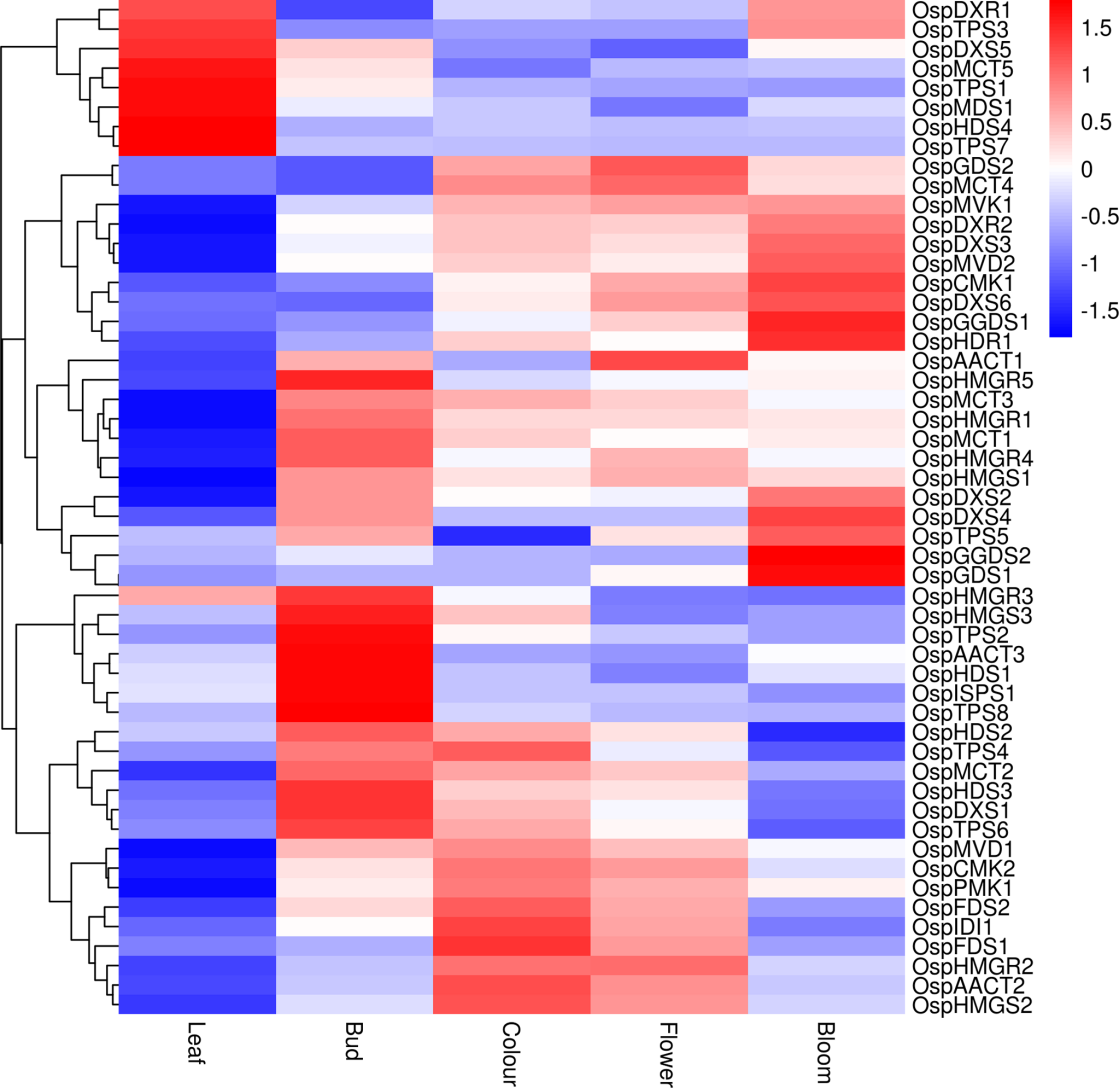
Heat map showing expression patterns of genes related to terpenoids in ‘Taihang Mingzhu’ leaf and inflorescences at different stages of development.

The DEGs associated with terpenoid content were screened from the transcriptomic data based on comparisons among different growth and developmental stages (**Figure 6**). The transcript levels of *OspDXR1, OspTPS3, OspDXS5, OspMCT5, OspTPS1, OspMDS1, OspHDS4*, and *OspTPS7* were higher in the leaf than in the inflorescences. The transcript levels of *OspHMGR3, OspHMGS3, OspTPS2, OspAACT3 OspHDS1, OspSPS1*, and *OspTPS8* were slightly higher in the inflorescences at the bud stage than in those at other stages. The transcript levels of *OspTPS4, OspIDI1*, and *OspFDS1* were higher in inflorescences at the color-development stage than in those at other stages, while that of *OspTPS5* was lower. The transcript level of *OspAACT* was higher in inflorescences at the flowering stage than in those at other stages. The transcript levels of *OspGGDS1, OspHDR1, OspGGDS2, OspGDS1*, and *OspGDS3* were higher in inflorescences at the full-bloom stage than in those at other stages. These results identify important candidate genes and provide a theoretical basis for further research on terpenoid biosynthesis in flowers of ‘Taihang Mingzhu’ at different developmental stages.

### 2.7 Verification of gene expression profiles

**Figure 7:**
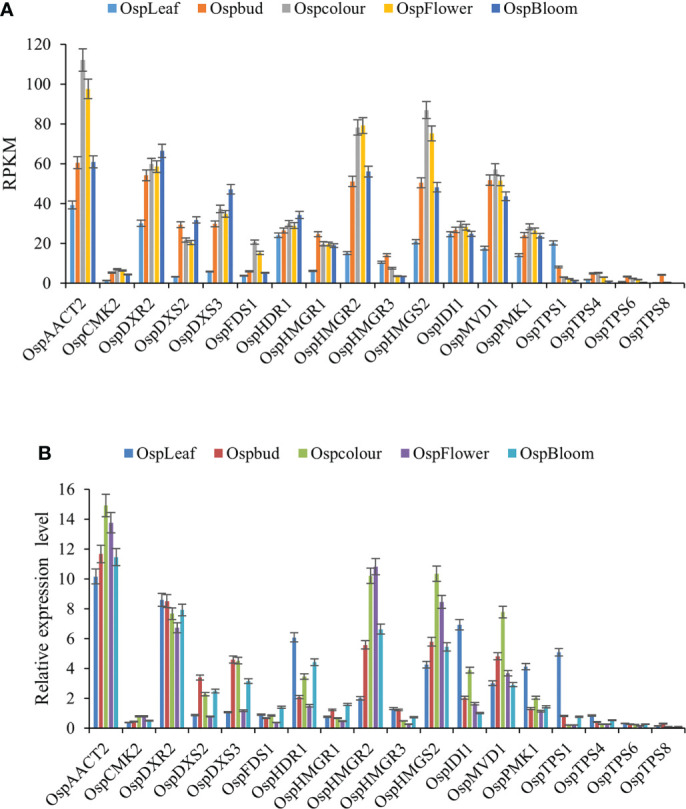
Transcript profiles of 18 selected genes in ‘Taihang Mingzhu’ as determined by qRT-PCR analysis. **(A)** Transcript levels of 18 genes based on reads per kb per million reads (RPKM) value. **(B)** Relative transcript levels of 18 genes as determined by qRT-PCR analysis. Error bars represent standard deviation from three replicates.

To further validate the transcript profiles of genes obtained from the Illumina sequencing analysis, 18 highly expressed and differentially paired unigenes were selected for qRT-PCR analyses. These genes encoded components of the terpenoid biosynthesis pathway. Their transcriptional patterns detected by qRT-PCR were similar to those detected from the transcriptome data (**Figure 7**). These results confirmed the reliability of the RNA-seq data, providing the basis for further analyses of the key genes involved in terpenoid synthesis in ‘Taihang Mingzhu’ (**Additional files 6**, **7**).

### 2.8 Weighted gene co-expression network analysis

**Figure 8:**
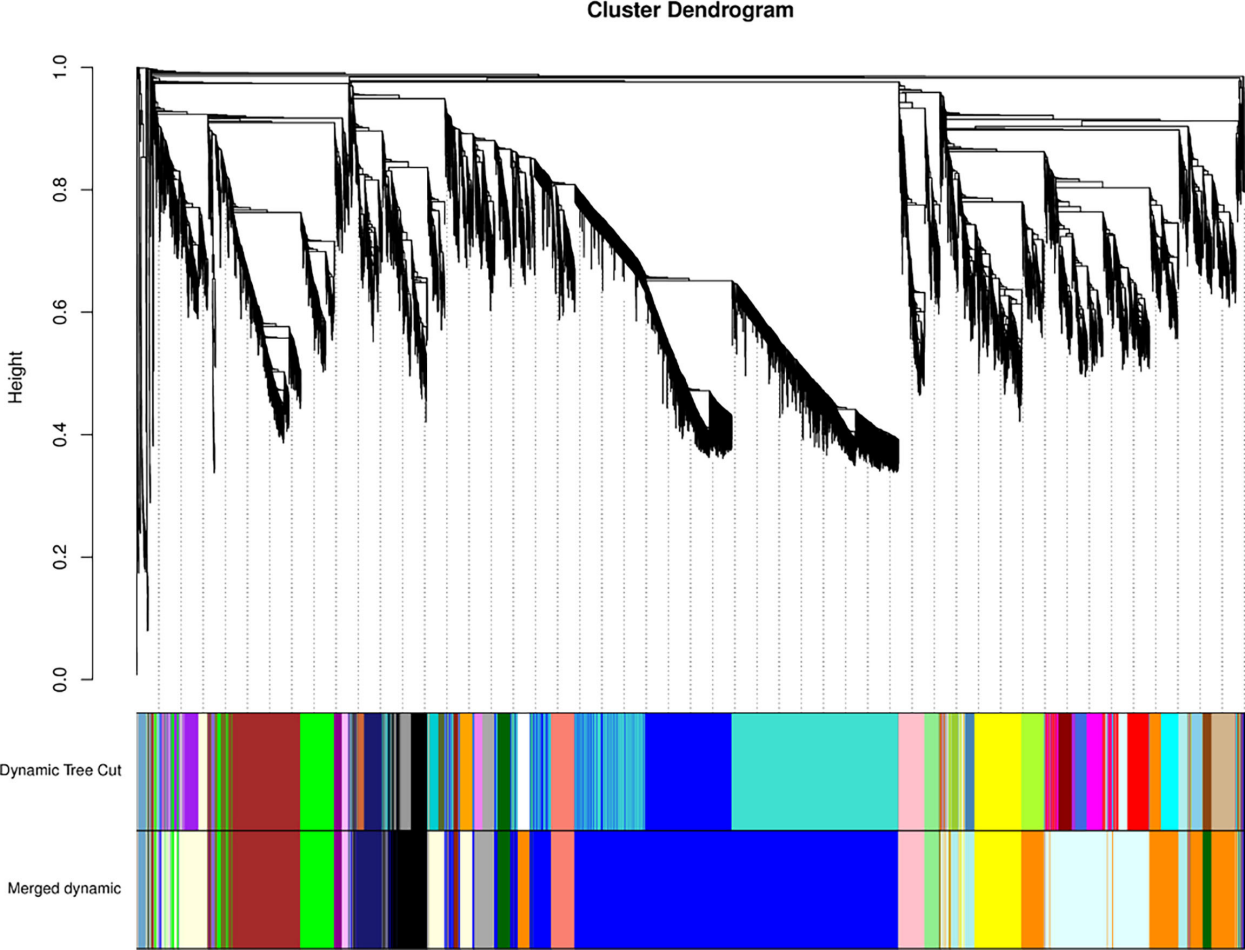
Modular tree of genes.

**Figure 9:**
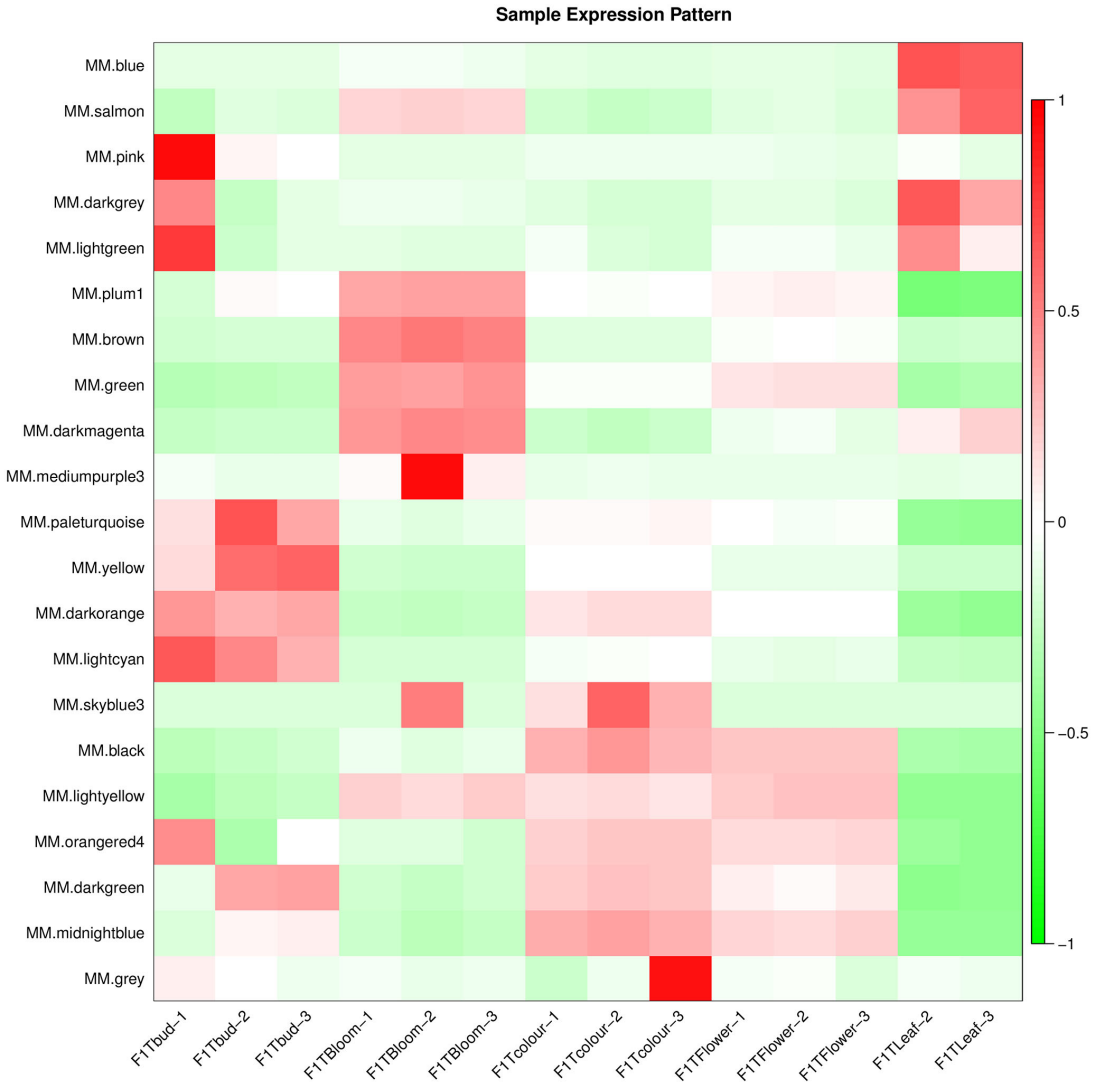
Gene expression patterns in different modules.

We conducted a weighted gene co-expression network analysis (WGCNA) analysis of the transcriptome data to identify gene modules that were highly co-expressed in both periods (Zhou et al., 2022). A module tree was constructed for modules with similar expression patterns based on the similarity of module eigenvalues (**Figure 8**). The WGCNA divided the genes into 21 modules, with different genes in each module. The blue module contained the most genes (5552 unigenes), followed by the lightcyan module (1709 unigenes), and then the darkorange module (1619 unigenes). The mediumpurple 3 module had the fewest genes (53 unigenes) (**Additional file 8**). The expression patterns of module genes in each sample are presented in terms of module eigenvalues. A heat map was constructed to show the gene expression patterns in different samples. Comparing gene expression among the samples (**Figure 9**), the leaf had higher module eigenvalues for the blue, salmon, and darkgrey modules; the inflorescence at the bud stage had higher module eigenvalues for the pink, lightgreen, darkorange, and lightcyan modules. The inflorescence at the color-development stage had higher module eigenvalues for the black, skyblue3, and midnightblue modules. The module eigenvalues were higher for the black and lightyellow modules in the inflorescence at the flowering stage; and higher for the plum1, brown, green, and darkmagenta modules in the inflorescence at the full-bloom stage. These results highlighted a large number of candidate genes involved in the molecular regulation of flowering and aromatic substance accumulation in ‘Taihang Mingzhu’.

### 2.9 Combined analyses of transcriptome and metabolome data

**Figure 10:**
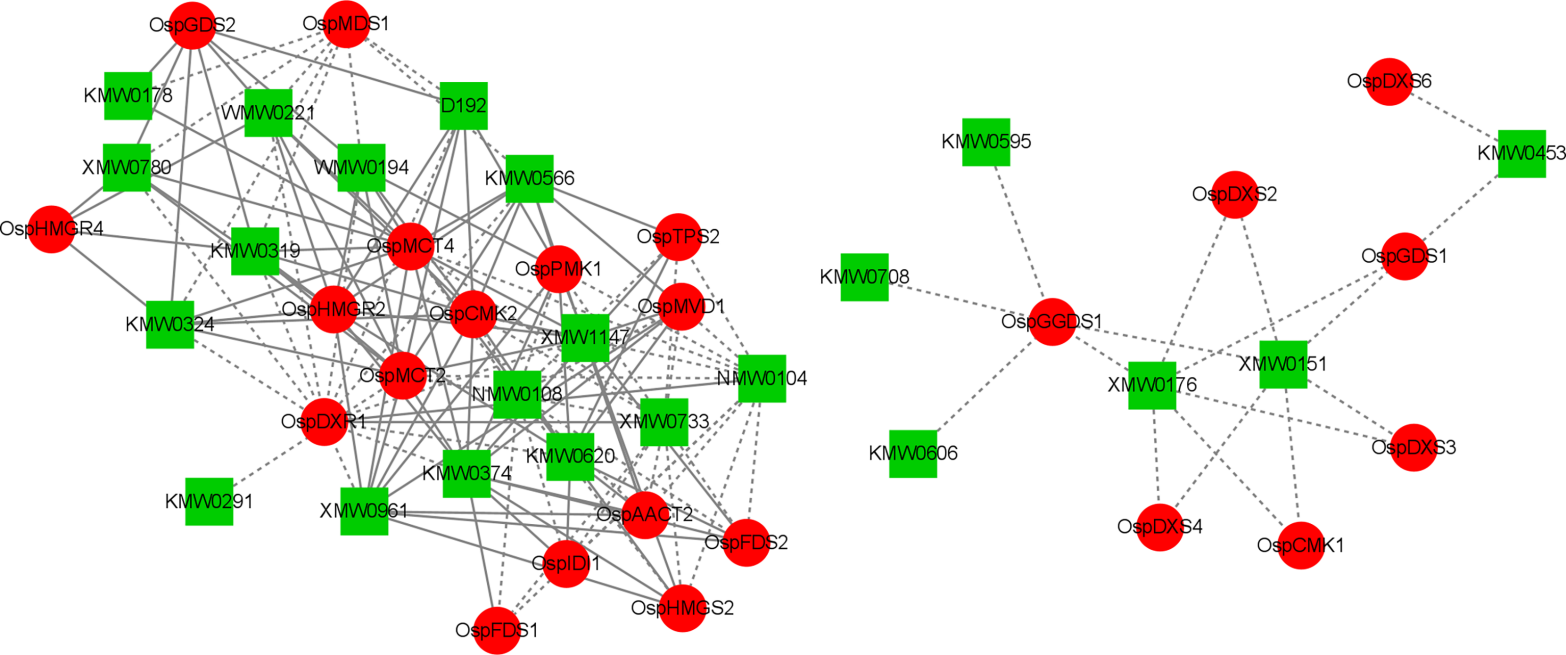
Correlation network diagram (red circles indicate genes; green rectangles indicate metabolites; solid lines indicate positive correlations; dashed lines indicate negative correlations).

To better understand the association between genes and terpenoids, we conducted correlation analyses to integrate the gene transcriptome and metabolite data. In these analyses, we selected correlations with Pearson’s correlation coefficient (PCC) > 0.8 and *P* < 0.05. Cytoscape 3.7.2 (Cytoscape Consortium, San Diego, CA, USA) was used to visualize and plot the correlation network of 52 terpene synthesis genes with the top 10 most abundant terpenes at each sampling time (Wang et al., 2022) (**Figure 10**, **Additional file 9**).

These comprehensive analyses revealed that many DEGs are involved in the terpenoid synthesis pathway. There were more positive correlations between different genes and different metabolites, with 87 groups positively correlated and 69 groups negatively correlated. Bornyl acetate (XMW0961) was positively correlated with nine DEGs and negatively correlated with one DEG; tricyclo[2.2.1.0(2,6)]heptane-3-methanol, 2,3-dimethyl- (XMW1147) was positively correlated with nine DEGs and negatively correlated with one DEG; 7-oxabicyclo[4.1.0]heptane-2-one, 3-methyl-6-(1-methylethyl)- (XMW0733) was negatively correlated with 11 DEGs and positively correlated with one DEG; epizonarene (XMW0151) was negatively correlated with six DEGs; benzene, 1-(1,5-dimethyl-4-hexenyl)-4-methyl- (KMW0620) was positively correlated with 10 DEGs and negatively correlated with one DEG; (4aR-trans)-decahydro-4a-methyl-1-methylene -7-(1-methylethylidene)-naphthalene (KMW0566) was positively correlated with eight DEGs and negatively correlated with two DEGs; 4-hexen-1-ol, 5-methyl-2-(1-methylethenyl)-, (R)- (KMW0374) was positively correlated with 12 DEGs and positively correlated with one DEGs. These results suggest that there is a complex regulatory relationship between gene expression and the accumulation of terpenoids.

### 2.10 Extraction and analysis of essential oil from leaves

Essential oil was extracted from the leaves by hydrodistillation. A compositional analysis of the essential oil revealed 270 metabolites (**Additional file 10**). A large proportion of these metabolites were terpenoids (76), followed by esters (50), heterocyclic compounds (39), aromatic hydrocarbons (19), alcohols (17), aldehydes (12), ketones (12), hydrocarbons (11), and halogenated hydrocarbons (9). Some of the less abundant metabolites were amines (8), nitrogenous compounds (6), acids (4), phenols (3), sulfur-containing compounds (3), and others (1). The most abundant aromatic substance was the terpenoid linalool, suggesting that this compound confers the special aroma of ‘Taihang Mingzhu’ leaves. These findings highlight that the leaves are a very good material for essential oil extraction, thereby improving the economic value of ‘Taihang Mingzhu’.

## 3 Discussion

In this study, we conducted transcriptomic and metabolomic analyses of the leaf and inflorescences of ‘Taihang Mingzhu’ at different developmental stages. Using high-quality RNA-Seq reads and assembly software, a total of 82,685 unigene sequences were assembled. The size of the assembly was 67,629,859 bp and the GC content was 39.2343%. The N50 was 1314 bp, the maximum read length was 13,054 bp, the minimum read length was 201 bp, and the average read length was 817 bp. The unigene N50 value was larger than the average read length, confirming the high quality of the assembly.

Through transcriptomic and metabolomic analyses of leaf, bud and inflorescences at different developmental stages, we identified 52 genes that are likely involved in regulating the synthesis of terpenoids that confer the aroma of ‘Taihang Mingzhu’. All terpenoids are produced from the C5 carbon precursors isopentenyl diphosphate (IPP) and dimethyl allyl diphosphate (DMAPP), and a range of terpenoids are produced in the presence of terpene synthases (TPSs) (Roeder et al., 2007). At present, most floral aroma research is focused on the genes involved in terpenoid synthesis. The plant TPSa family is a medium-sized gene family divided into seven subfamilies; TPS-a to TPS-h (Chen et al., 2011). Usually, the more terpenoids a plant releases, the more TPSs it contains (Myburg et al., 2014). TPS families have been identified in many plants whose genomes have been fully sequenced. In *Albizia julibrissin*, five TPSs are involved in the synthesis of volatile terpenoids. γ-terpinene and 1R-α-pinene produced by AjTPS9 are components of floral and leaf volatiles, respectively (Liu et al., 2021). Moreover, *TPS* genes have been identified in ornamental plants such as rose and goldenseal (Guterman et al., 2002; Nagegowda et al., 2008). A total of eight homologous *TPS* genes were among those identified as being related to the synthesis of terpenoid aroma compounds in ‘Taihang Mingzhu’. Among them, *OspTPS1* and *OspTPS7* showed higher transcript levels in the leaves than in the inflorescences; *OspTPS2* and *OspTPS8* showed higher transcript levels in inflorescences at the bud stage than in those at other stages; and *OspTPS4* showed higher transcript levels at the color-development stage than at the other stages of inflorescence development. These genes are important candidate gene resources for molecular biology studies and molecular breeding to manipulate terpenoid aromatics in *Opisthopappus* Shih.

A total of 1350 aromatic metabolites were detected in the leaves and inflorescences of ‘Taihang Mingzhu’ by HS-SPME-GC/MS, among which 302 were terpenoids. Of the 16 classes of aromatic substances detected, terpenoids were the most abundant, consistent with their roles as important components of the aroma of ‘Taihang Mingzhu’.

We detected 1235, 1274, 1271, and 1267 aromatic metabolites in the leaf and inflorescences at the color-development, flowering, and full-bloom stages, respectively, of which 292, 286, 286, and 286 were terpenoids. Leaves contained more terpenoids than did inflorescences. Sixteen terpenoids were specifically synthesized in the leaf: 1,3,6-octatriene, 3,7-dimethyl-, (Z)-; beta-ocimene; 1,5-heptadien-4-ol, 3,3,6-trimethyl-; 2,4,6- octatriene, 2,6-dimethyl-; 2-cyclohexen-1-one, 3-methyl-6-(1-methylethyl)-; (-)-carvone; (4R,4aS,6S)-4,4a-dimethyl-6-(prop-1-en-2-yl)-1,2,3,4,4a,5,6,7-octahydronaphthalene; 1,1,7,7a-tetramethyl-1a,2,6,7,7a,7b-hexahydro-1H-cyclopropa[a]naphthalene; naphthalene,1,2,4a, 5,6,8a-hexahydro-4,7-dimethyl-1-(1-methylethyl)-,[1R-(1.alpha.,4a.alpha.,8a.alpha.)]-; spiro[4.5]dec-7-ene, 1,8-dimethyl-4-(1- methylethenyl)-, [1S-(1.alpha.,4.beta.,5.alpha.)]-; aromandendrene; carvenone; 2,6-octadienal, 3,7-dimethyl-, (E)-; cyclohexene, 3-(1,5-dimethyl-4-hexenyl)-6-methylene-, [S-(R*,S*)]-; isospathulenol; and hibaene. Quantitative metabolite analyses revealed that the relative content of terpenoids was highest in the leaf (46.13%), followed by inflorescences at the flowering stage (38.78%), color-development stage (38.19%), and full-bloom stage (37.66%). The most abundant terpene in the leaf was linalyl acetate, a monoterpene with a sweet fruity floral aroma that is formed mainly by acetylation of linalool. Linalyl acetate is mainly found in the essential oils of plants such as lavender (Miastkowska et al., 2021) and clary sage (Schmiderer et al., 2010). Linalyl acetate has anti-inflammatory (Peana et al., 2002) and antioxidant properties (Ziaee et al., 2015), and has a therapeutic effect against diabetes (Shin et al., 2018). It is also used in cosmetics, perfumes, shampoos, and daily products due to its unique aroma, and in small quantities in edible flavors.

The qualitative and quantitative analyses of the compounds in ‘Taihang Mingzhu’ revealed that the leaf contained the most terpenoids. Moreover, leaves can be collected in three seasons each year—spring, summer, and autumn—with large yields. Therefore, the leaves are an important resource for the extraction of essential oil. This adds value to the leaves, which in turn increases the economic value of *Opisthopappus* Shih.

We analyzed the aromatic substances in the inflorescences of ‘Taihang Mingzhu’ at different developmental periods. In total, we detected 286 terpene metabolites in inflorescences at the color-development, flowering, and full-bloom stages, but their contents varied among the different stages. The proportions of terpenoids out of total aromatic substances in ‘Taihang Mingzhu’ inflorescences at the color-development, flowering, and full-bloom stages were 38.19%, 38.78%, and 37.66%, respectively. Ten terpenoids were specific to the inflorescences: bicyclo[3.1.1]heptan-3-ol, 6,6-dimethyl-2-methylene-; cyclohexanol, 1-methyl-4-(1-methylethyl)-, cis-; 2H- pyran, 3,6-dihydro-4-methyl-2-(2-methyl-1-propenyl)-; p-mentha-1,5,8-triene; 2,6-dimethyl-1,3,5,7-octatetraene, E,E-; bicyclo[7.2.0] undec-4-ene, 4,11,11-trimethyl-8-methylene-,[1R-(1R*,4Z,9S*)]-; bornyl acetate; 2-cyclohexen-1-one, 2-hydroxy-3-methyl-6-(1-methylethyl)-; tricyclo[2.2.1.0(2,6)]heptane-3-methanol, 2,3-dimethyl-; and cyclopropanecarboxylic acid, 2,2-dimethyl-3-(2-methyl-1-propenyl)-, (1R- trans)-. Inflorescences at the color-development stage had highest number of aromatic compounds, indicating that this is the optimal stage for harvest.

Terpenoids are the largest class of plant secondary metabolites and are associated with a wide range of biological activities (Tholl, 2015). The strength of antimicrobial activity is related to the presence of hydroxyl groups (phenolic and alcoholic compounds). Oxyterpenes have strong antibacterial activity against all bacteria. For example, eugenol has a rapid antibacterial effect against *Salmonella* serovar Typhimurium, pineol has a good antibacterial effect against *Staphylococcus aureus*, and citronellol and geraniol have rapid antibacterial effects against *Escherichia coli* (Guimarães et al., 2019). All of these bacteria are microorganisms that affect food quality and safety (Walkeryorkmoore et al., 2017). The antibacterial properties of terpenoids are exploited to ensure food safety. Meroterpenoid compounds derived from β-pineolene act as potent α-glucosidase inhibitors and are being used in the development of anti-diabetic drugs (Ma et al., 2018). Treatment of wounds with α-pineolene and α-watercressene results in stress-resistant scars on the wound surface and accelerates wound contraction, suggesting that α-pinene and α-apigenin have some wound-healing activity (Salas-Oropeza et al., 2021).Terpenoids such as limonene, linalool, β-caryophyllene, and α-pinene extracted from cannabis have anti-inflammatory activity (Downer, 2021). Artemisinins (sesquiterpenoids) obtained from *Artemisia annua* have anti-malarial activity, and therefore have potential uses as pharmaceuticals (Chandramouli et al., 2022). Lauricene, β-staphylene, and linalool in the volatile oil of hops are commonly used as anticancer drugs (Miyashita and Sadzuka, 2013). Because terpenoids have a diverse range of biological activities, it is important to study their composition and biosynthesis. Therefore, the metabolic pathways of terpenoids and their gene regulatory networks in ‘Taihang Mingzhu’ were analyzed to reveal the correlation between particular genes and terpenoids.

## 4 Conclusion

We used a combination of HS-SPME-GC/MS and RNA-seq analyses to detect metabolites and genes showing differences in abundance among the leaf and flowers of ‘Taihang Mingzhu’ at different developmental stages. The results showed that the leaves of ‘Taihang Mingzhu’ are a good resource for essential oil extraction, which enhances the economic value this plant. We analyzed the metabolic pathways of terpene metabolites and gene regulatory networks during the development of ‘Taihang Mingzhu’ inflorescences. We identified 52 regulatory genes related to terpene synthesis. The active expression of these genes resulted in the synthesis and accumulation of a rich variety of terpenoids in the inflorescences at the color-development, flowering, and full-bloom stages, as well as in the leaf. This study is the first to analyze the gene regulatory network of terpene metabolites in *Opisthopappus* Shih. The identification of candidate genes regulating terpene metabolite synthesis provides a basis for further research on aromatic substances. These findings also lay an important theoretical foundation for further studies, and identify genetic resources for molecular biology research and breeding of endangered *Opisthopappus* Shih in China.

## 5 Materials and methods

### 5.1 Transcriptome approach

#### 5.1.1 Plant materials and RNA extraction

‘Taihang Mingzhu’ is a hybrid of *O. taihangensis.* To obtain ‘Taihang Mingzhu’ stamens were removed from the flowers of the mother plant *O. taihangensis* and the flowers were left unbagged. The flowers were pollinated naturally by bees, with pollen from wild species of *Chrysanthemum indicum* var. aromaticum, *Chrysanthemum dichrum*, *Chrysanthemum lavandulifolium*, and some small *chrysanthemum* species that were cultivated in the same greenhouse. The seeds were harvested from the mother plant *O. taihangensis* and sown. Superior plants with high fragrance and ornamental value were selected after flowering the following year.

Cuttings of ‘Taihang Mingzhu’ were cultivated in a greenhouse at the Shunyi base of Beijing Liu Wenchao Institute of Summer Chrysanthemums Breeding Science and Technology (40°149633′N,116°619353′E). The duration from planting cuttings to flowering was about 120 days. About 10–20 buds and inflorescences at the color-formation, flowering, and full-bloom stages and leaves of ‘Taihang Mingzhu’ were collected between 7 a.m. to 10 a.m., with three biological replicates for each sample. The collected samples were immediately frozen in liquid nitrogen and stored at −80°C for RNA sequencing and metabolic profiling analyses. Total RNA was extracted from the above samples using the RNeasy Plant Mini Kit (Qiagen, Hilden, Germany). The RNA concentration was determined using a NanoDrop ND2000 spectrophotometer (Thermo Scientific, Waltham, MA, USA).

#### 5.1.2 Data filtering and unigene assembly

The raw data were subjected to several filtering steps to ensure quality and reliability. This included removing reads containing adapters, those with N>10% (where N is an unknown base), and low-quality reads (reads with Qphred <= 20 bases accounting for more than 50% of the entire read length). All subsequent analyses were performed using high-quality clean data. The clean reads were spliced using Trinity. The longest contig sequence was used as the unigene in subsequent analyses after hierarchical clustering of contigs.

#### 5.1.3 Library construction and sequencing

After extracting total RNA, eukaryotic mRNA was enriched with oligo(dT) beads, while prokaryotic mRNA was enriched by removing rRNA with the Ribo-Zero™ Magnetic Kit (Epicentre Biotechnologies, Madison, WI, USA). The enriched mRNA was then cut into short fragments with fragmentation buffer and reverse-transcribed into cDNA with random primers. cDNA was synthesized using DNA polymerase I, RNase H, dNTP, and buffer to synthesize second-strand cDNA. cDNA fragments were then purified with a QiaQuick PCR extraction kit, end-repaired, added to poly(A), and ligated to Illumina sequencing adapters. The size of the ligated product was selected by agarose gel electrophoresis. The products were PCR-amplified and then sequenced on the Illumina HiSeq™ 4000 platform by Gene Denovo Biotechnology Co. (Guangzhou, China).

#### 5.1.4 Basic annotation of unigenes

The basic annotation of unigenes included protein function annotation, pathway annotation, COG/KOG functional annotation, and Gene Ontology (GO) annotation. To annotate unigenes, we use the Blastx program (http://www.ncbi.nlm.nih.gov/BLAST/) to search sequences against the NCBI non redundant protein (NR) database (http://www.ncbi.nlm.nih.gov), Swissprot protein database (http://www.expasy.ch/sprot), Kyoto Encyclopedia of Genes and Genomes (KEGG) database (http://www.genome.Jp/kegg), and the COG/KOG database (http://www.ncbi.nlm.nih.gov/COG). The annotation was assigned on the basis of the best comparison results.

#### 5.1.5 Gene expression levels and differentially expressed genes analysis

The unigene transcript levels were normalized to RPKM (reads per kb per million reads) values. To verify the *p*-value threshold, the false discovery rate (FDR) was estimated in several tests and analyses (Endress, 2006). The thresholds for determining whether gene expression differences were significant were: log2(ratio) ≥ 2 in absolute value and FDR < 0.05 (Mortazavi et al., 2008). The DEGs were those showing at least a 2-fold difference in transcript levels between compared samples.

#### 5.1.6 Determination of gene transcript levels by qRT -PCR

For qRT-PCR assays, total RNA was extracted from each tissue of ‘Taihang Mingzhu’ individually and treated with DNase (Promega, Madison, WI, USA) to eliminate residual genomic DNA. Purified RNA was used as template for cDNA synthesis using a reverse transcription kit (Tsingke, Beijing, China). cDNA was synthesized using the PikoReal system (Thermo Fisher Scientific) for qRT-PCR analyses. Each reaction was performed with 2 μL of cDNA as template in a total volume of 20 μL. The PCR program was as follows: 95°C for 5 s; 54°C for 30 s, and 72°C for 30 s for 39 cycles. Three biological replicates were analyzed for each sample. The *C. morifolium* protein phosphatase 2A (PP2Ac) gene was used as a reference control and the 2-ΔΔCt method was used to calculate the relative gene transcript levels (Zhou et al., 2020).

### 5.2 Metabolomic methods

#### 5.2.1 Sample preparation and extraction

The samples were removed from storage at −80°C, ground in liquid nitrogen, and then the homogenate was vortexed and mixed well. For each sample, about 500 mg (1 mL liquid) was added to a headspace vial (Agilent, Palo Alto, CA, USA) containing saturated NaCl solution. Then, 10 μL (50 μg/mL) internal standard solution was added. Fully automated HS-SPME was performed to extract compounds for GC-MS analysis (Yuan et al., 2022).

#### 5.2.2 Gas chromatography - mass spectrometry analyses

After sampling, volatile organic compounds (VOCs) in the fiber coating were desorbed at the inlet of a gas chromatograph (Model 8890; Agilent) in splitless mode for 5 min at 250°C. The VOCs were separated using a Agilent Model 8890 gas chromatograph on a 30 m × 0.25 mm × 0.25 µm DB-5MS (5% phenyl-polymethylsiloxane) capillary column. The GC was connected to a 7000D mass spectrometer (Agilent). Helium was used as carrier gas with a linear velocity of 1.2 mL/min. The injector temperature was 250°C and the detector temperature was 280°C. The oven temperature started at 40°C (3.5 min) and increased at 10°C/min to 100°C, then at 7°C/min to 180°C, and then at 25°C/min to 280°C, where it was held for 5 min. Mass spectra were recorded in the electron impact (EI) ionization mode at 70 eV. The temperatures of the quadrupole mass detector, ion source, and transmission line were set to 150°C, 230°C, and 280°C, respectively. The mass spectra collected in selected ion monitoring (SIM) mode were used for analyte identification and quantification.

#### 5.2.3 Principal component and cluster analyses

Unsupervised principal PCA was performed using the statistics function prcomp in R (www.r-project.org) (Chen et al., 2009). The metabolite content data were subjected to unit variance scaling before unsupervised PCA. The heat map was drawn using the ComplexHeatmap package in R. The hierarchical cluster analysis (HCA) revealed the patterns of metabolite accumulation among different samples (Yu et al., 2012).

#### 5.2.4 Combined transcriptome and metabolome analyses

We performed Pearson’s correlation analyses to detect relationships between gene transcript levels and metabolite contents. Pearson’s correlation coefficient (PCC) values of > 0.8 and *P* < 0.05 were selected and visualized using Cytoscape 3.7.2 (Cytoscape Consortium, San Diego, CA, USA).

#### 5.2.5 Screening for differentially accumulated metabolites

An OPLS-DA was performed using the OPLSR.Anal function of the MetaboAnalystR package in R. Based on the OPLS-DA results, the VIP values from the multivariate OPLS-DA model were used for preliminary screening of metabolites that differed among tissues (Thévenot et al., 2015). The *p*-value and FC value from the univariate analysis were combined with the *p*-value of the univariate analysis to further screen for differentially accumulated metabolites. If the number of biological replicates is < 3, differences can be detected based on the FC value.

### 5.3 Extraction of essential oils from leaves

The leaves of ‘Taihang Mingzhu’ were placed in a distillation apparatus, and distilled water was added at a ratio of 1:2. The distillation process continued until no more essential oil separated from the leaves. The essential oil was collected and stored at 4°C until analysis.

## Data availability statement

The data presented in the study are deposited in the NCBI Sequence Read Archine, BioProject accession number: PRJNA868887 and SRA accession number: SRR21075407-SRR2107542116.

## Author contributions

HL, WC, YC, HC, and XT conducted the experiments. WC analyzed the data and prepared the manuscript. HL reviewed the manuscript. WL, CL, DC, XC, LS, FW, and CH provided assistance. All authors read and approved the final manuscript.

## Funding

This work was supported by Beijing Innovation Consortium of Agriculture Research System (BAIC09-2022), the National Natural Science Foundation of China (31901354), the Innovation Foundation of the Beijing Academy of Agriculture and Forestry Sciences (KJCX20200112), and Special fund for reform and development “Evaluation of volatile flavor components in food and establishment of fingerprint” (GGFA292206).

## Acknowledgments

We thank Jennifer Smith, PhD, from Liwen Bianji (Edanz) (www.liwenbianji.cn/) for editing the English text of drafts of this manuscript.

## Conflict of interest

The authors declare that the research was conducted in the absence of any commercial or financial relationships that could be construed as a potential conflict of interest.

## Publisher’s note

All claims expressed in this article are solely those of the authors and do not necessarily represent those of their affiliated organizations, or those of the publisher, the editors and the reviewers. Any product that may be evaluated in this article, or claim that may be made by its manufacturer, is not guaranteed or endorsed by the publisher.

## Supplementary material

Additional file 1 Leaves.

Additional file 2 Metabolite summary table.

Additional file 3 Differentially accumulated metabolites among groups.

Additional file 4 KEGG pathways enriched with unigenes.

Additional file 5 Terpenoid synthesis-related genes.

Additional file 6 Gene fluorescence quantification primer sequences.

Additional file 7 Correlation between RT-qPCR and RPKM results of gene transcript levels.

Additional file 8 Number of genes in the module.

Additional file 9 Genes and metabolites used in the correlation network map.

Additional file 10 Metabolites in ‘Taiyang Mingzhu’ essential oil.

## Abbreviations

AACT, acetoacetyl-CoA thiolase; AcAc-CoA, acetoacetyl-CoA; CMK, CDP-ME kinase; DEGs, differentially expressed genes; DMAPP, dimethylallyl diphosphate; DOXP, 1-deoxy-D-xylulose 5-phosphate; DXS, DOXP synthase; DXR, DOXP reductoisomerase; FDS, farnesyl diphosphate synthase; FPP, farnesyl diphosphate; GA-3P, glyceraldehyde-3-phosphate; GC-MS, gas chromatography-mass spectrometry; GDS, geranyl diphosphate synthase; GGDS, geranyl geranyl diphosphate synthase; GGPP, geranyl geranyl diphosphate; GGPPS, geranyl geranyl diphosphate synthase; GPP, geranyl diphosphate; HDR, (E)-4-hydroxy-3-methylbut-2-enyl diphosphate reductase; HDS, (E)-4-hydroxy-3-methylbut-2-enyl diphosphate synthase; HMBPP, (E)-4-hydroxy-3-methylbut-2-enyl diphosphate; HMG-CoA, 3-hydroxy-3-methylglutaryl-CoA; HMGR, HMG-CoA reductase; HMGS, HMG-CoA synthase; HS-SPME, headspace solid-phase microextraction; IPP, isopentenyl diphosphate; MCT, 2-C-methyl-D-erythritol 4-phosphate cytidylyltransferase; MDS, 2-C-methyl-D-erythritol 2,4-cyclodiphosphate synthase; MEP, 2-C-methyl-D-erythritol 4-phosphate; MVA, mevalonate; MVA-5-phosphate, mevalonate-5-phosphate; MVD, mevalonate diphosphate decarboxylase; MVK, mevalonate kinase; OPLS-DA, orthogonal partial least squares discriminant analysis; PCA, principal component analysis PMK, phosphomevalonate kinase; PCR, polymerase chain reaction; qRT-PCR, quantitative real-time PCR; TPS, terpene synthase
