# Supplementary figures and images for: Terpenoids and their gene regulatory networks in Opisthopappus taihangensis ‘Taihang Mingzhu’ as detected by transcriptome and metabolome analyses
Source: Front Plant Sci. 2022 Sep 29;13:1014114. doi: 10.3389/fpls.2022.1014114 (PMC9557748; doi:10.3389/fpls.2022.1014114)

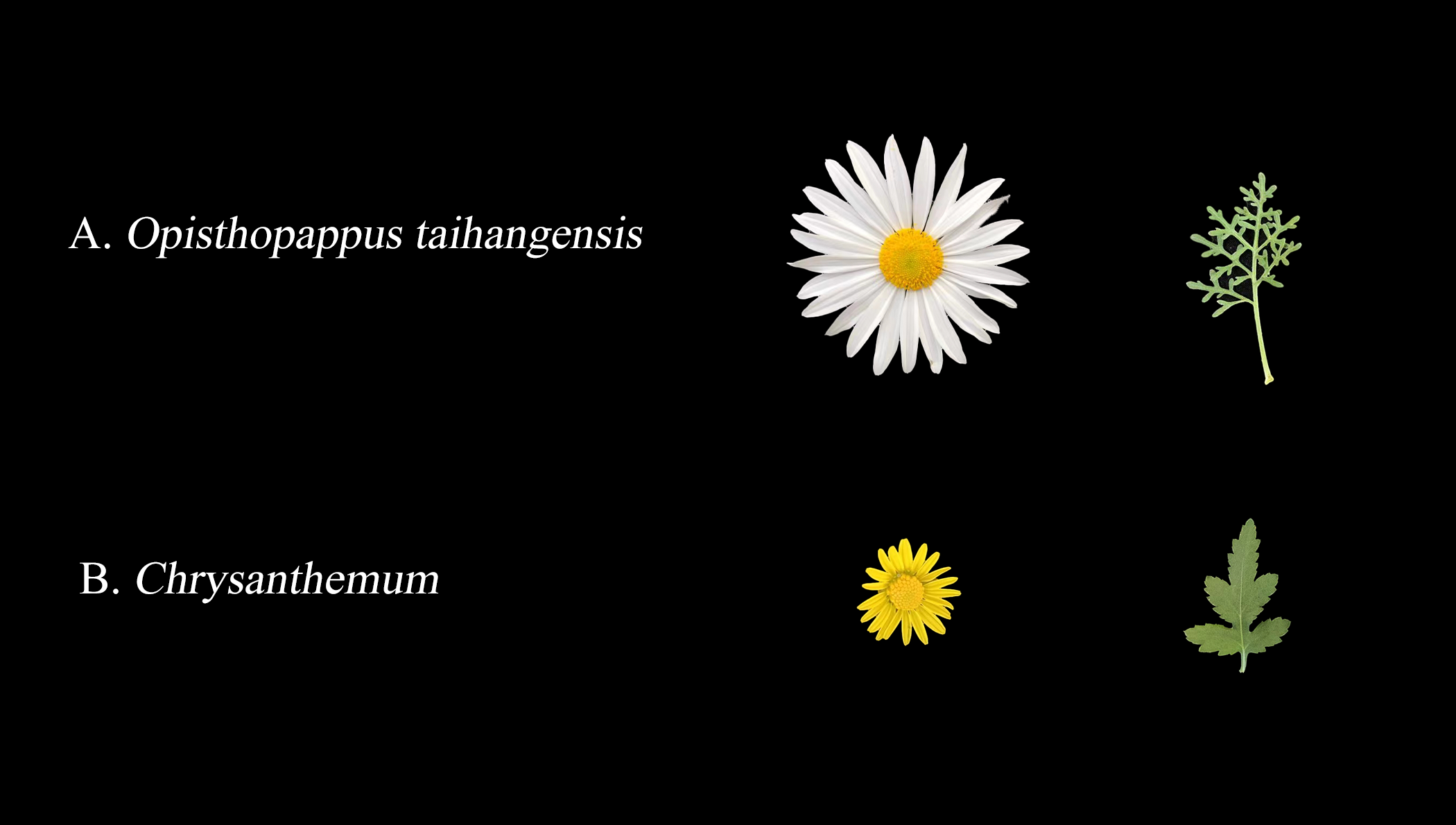

Supplement: Additional file 1 — Leaves. [file DataSheet_1.zip › Image 1.TIF]

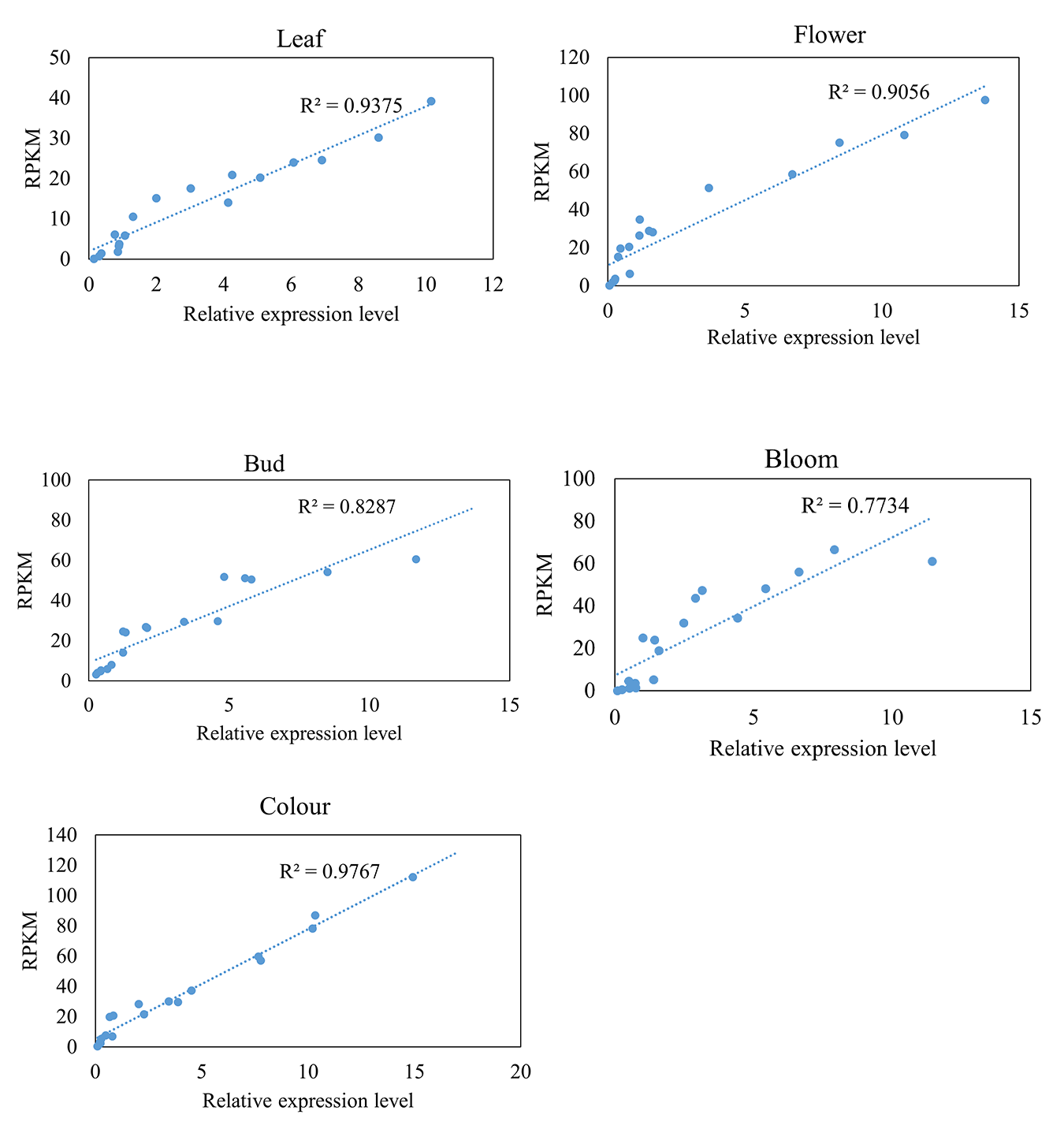

Supplement: Additional file 1 — Leaves. [file DataSheet_1.zip › Image 2.TIF]
